# Post-Autologous (ASCT) Stem Cell Transplant Therapy in Multiple Myeloma

**DOI:** 10.1155/2014/652395

**Published:** 2014-11-24

**Authors:** Zeina Al-Mansour, Muthalagu Ramanathan

**Affiliations:** Division of Hematology/Oncology, School of Medicine, University of Massachusetts, 55 Lake Avenue North, Worcester, MA 01655, USA

## Abstract

Autologous stem cell transplant (ASCT) is the standard of care in transplant-eligible multiple myeloma patients and is associated with significant improvement in progression-free survival (PFS), complete remission rates (CR), and overall survival (OS). However, majority of patients eventually relapse, with a median PFS of around 36 months. Relapses are harder to treat and prognosis declines with each relapse. Achieving and maintaining “best response” to initial therapy is the ultimate goal of first-line treatment and sustained CR is a powerful surrogate for extended survival especially in high-risk multiple myeloma. ASCT is often followed by consolidation/maintenance phase to deepen and/or maintain the response achieved by induction and ASCT. Novel agents like thalidomide, lenalidomide, and bortezomib have been used as single agents or in combination. Thalidomide use has been associated with a meaningful improvement in PFS and EFS, however, with substantial side effects. Data with lenalidomide maintenance after-ASCT is favorable, but the optimal duration of lenalidomide maintenance is still unclear. Bortezomib use has been associated with superior outcomes, predominantly in high-risk myeloma patients. Combination regimens utilizing a proteasome inhibitor (i.e., bortezomib) with an immunomodulatory drug (thalidomide or lenalidomide) have provided the best outcomes. This review article serves as a review of the best available evidence in post-ASCT approaches in multiple myeloma.

## 1. Introduction

Autologous stem cell transplant (ASCT) is the standard of care in transplant-eligible patients, based on several phase III trials and meta-analyses in the mid-1990s showing significant improvement in progression-free survival (PFS) and complete remission rates (CR) as well as overall survival (OS) [1–3]. In their IFM-90 trial, Attal et al. reported superior outcomes of high-dose chemotherapy (HDC) followed by ASCT compared to conventional chemotherapy alone, with improvement noted in OS (57%), complete remission rate CR (22%) and event-free survival EFS (16%), as well as median OS improvement to 57 months versus 44 months [1]. However, majority of patients eventually relapsed with a median PFS of around 36 months. Unfortunately, relapses are harder to treat and prognosis declines with each relapse. Therefore, achieving and maintaining “best response” to initial therapy is the ultimate goal of first-line treatment. Several studies have shown that sustained CR for 3 years or more from treatment initiation is a powerful surrogate for extended survival especially in high-risk multiple myeloma [4].

The treatment algorithm for newly diagnosed multiple myeloma (MM) has evolved over the last two decades with the incorporation of novel agents in myeloma induction regimens prior to ASCT [5, 6]. Improved outcomes when used before ASCT were the basis for the use of these agents in the post-ASCT setting as a means to* deepen* and* maintain* the response achieved by HDC-ASCT in myeloma patients. The benefit of maintenance therapy is thought to be due to suppression of clonal proliferation of myeloma cancer cells thereby delaying or preventing relapse.

In this review article, we will briefly review the significance of the depth of response to induction chemotherapy and will focus on how to deepen or maintain response by means of a consolidation approach, maintenance, or both.

## 2. Significance of Depth of Response to Chemotherapy

**Figure 1:**
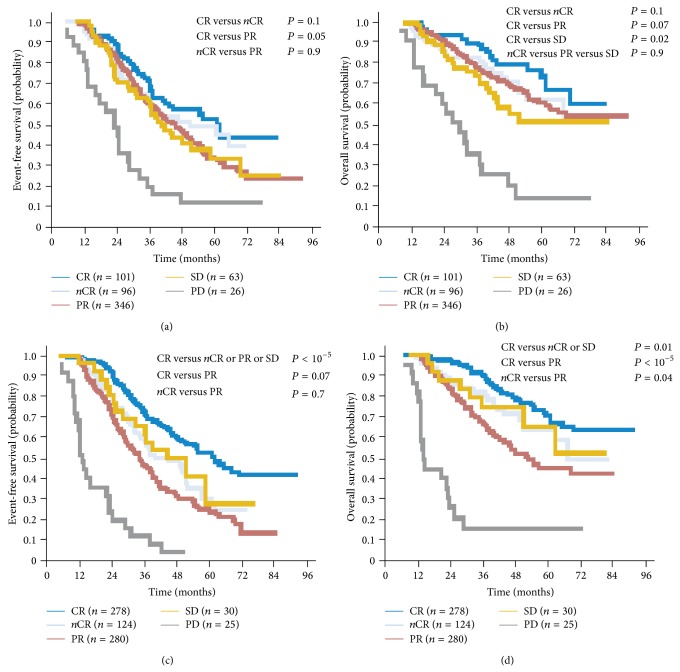
Achievement of CR is associated with improvement in survival outcomes after induction as well as after ASCT (adapted with permission from [10]).

Multiple studies have suggested a survival advantage from attaining a deeper response to induction chemotherapy and HDC-ASCT, and sustained CR was shown to be a surrogate for OS in myeloma population [7–14] (see Figure 1). In a meta-analysis of 21 studies including 4,990 patients in 10 prospective and 11 retrospective studies, highly significant associations between maximal response and survival outcomes were demonstrated [7]. However, it remains uncertain whether this just reflects underlying disease biology (i.e., prognostic marker) versus a specific treatment effect, especially since the definition of CR was historically ununified until the development of the IMWG treatment response criteria [8, 9].

**Figure 2:**
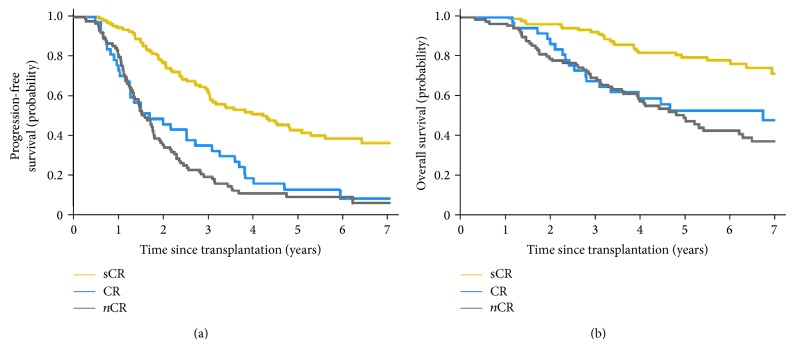
sCR is associated with superior PFS and OS compared to CR (adapted from [11]).

Furthermore, achieving stringent CR (sCR), as defined by IMWG criteria, is associated with even better outcomes compared to CR, with median time-to-progression of 50 months with sCR versus 20 months with CR [11, 12]. The benefit is more pronounced when comparing OS outcomes (see Figure 2).

### 2.1. Evaluation of Minimal Residual Disease (MRD)

The techniques for assessing disease burden in MM have evolved over time. Sensitive methods to detect MRD have been used to evaluate either immunophenotypic response or molecular response [13, 14, 18, 19]. In their recent article about* Controversies in the Assessment of Minimal Residual Disease in MM*, Corradini et al. reviewed the clinical significance of MRD negativity using highly sensitive techniques and concluded that, at the present time, the goal of assessing MRD is for risk stratification and to evaluate response to novel agents particularly in the context of clinical trials [20].

Immunophenotypic response assessment by multiparameter flow cytometry (MFC) was evaluated after ASCT in a subset of patients enrolled in Myeloma Research Council (MRC) IX, the Spanish GEM 2000, and GEM 2005 trials. Absence of MRD at D + 100 was associated with statistically significant improvement in both PFS and OS in patients with favorable and adverse cytogenetics [13, 21, 22]. Additionally, persistent MRD by MFC at D + 100 (HR 8.0, *P* = 0.005) and high-risk cytogenetics by FISH (HR 17.3, *P* = 0.002) were the only independent factors that predicted unsustained CR and shortened OS [22].

Molecular response can be assessed by either PCR-based techniques (either fluorescent or allele-specific oligonucleotide PCR) or next generation sequencing (NGS). MRD assessment by PCR was evaluated in several trials and homogenously showed that lower levels of MRD were associated with markedly better survival and longer disease-free periods [14, 23–25].

It should be noted, though, that these results were based on subsets of patients enrolled in the cited trials where MRD assessment was done only in those who had a bone marrow sample at D + 100 (in those assessed by MFC), thus raising concern for selection bias. Additionally, variety of technical factors can affect the sensitivity, specificity, and applicability of MFC such as time of sampling with respect to treatment, number of markers, number of cells counted, and marrow cellularity. On the other hand, PCR and NGS, despite being very sensitive for MRD detection, are limited by the technical complexity and the lack of validated threshold to separate high from low MRD to better allow establishment of prognostic variables [21–26].

In conclusion, all methods reliably differentiated between MRD-positive versus -negative cases and successfully correlated MRD negativity with improved outcomes. MFC appears to have the greatest applicability, whereas PCR and NGS demonstrated higher sensitivity. Other techniques for MRD assessment such as detection of circulating tumor cells and whole-body PET-CT/MRI seem to have potential to add sensitivity for MRD detection, especially in extramedullary disease and/or oligo- or nonsecretory MM [26]. However, these approaches are still investigational and need further validation in the context of clinical trials.

## 3. Consolidation and Maintenance Strategies in MM

Despite the remarkable improvement in the outcome of MM treatment over the last two decades with the use of immunomodulatory and novel agents, MM remains an incurable disease. Multiple studies over the last few years have examined the role of maintenance, consolidation, or both to eliminate residual disease after HDC-ASCT in MM. Consolidation is typically a short course, more intensive, with the main goal of deepening the response achieved by induction chemotherapy and HDC-ASCT. Maintenance is given for a prolonged period of time with the goal of preventing/delaying disease progression. Both approaches showed favorable outcomes in terms of delaying relapse and need for second line intensive therapy. However, it remains unclear whether there is a significant survival advantage to justify the cost and toxicity of continued treatment. The ideal regimen should be easy to deliver, convenient to use, cost-effective, have modest toxicity and lead to improved PFS and ideally OS over retreatment at relapse [27, 28].

Historically, interferons (IFN) and glucocorticoids were the first agents studied in the maintenance setting after induction. Berenson et al. compared alternate-day oral prednisone at 2-dose levels (10 mg versus 50 mg) in a subset of patients achieving at least 25% tumor reduction following induction therapy during their enrollment in SWOG 9210 trial (*n* = 125). Significant improvements were noted in both PFS (14 versus 5 months, *P* = 0.003) and OS (37 versus 26 months, *P* = 0.05) favoring those receiving the 50 mg dose [29].

## 4. Post-ASCT Strategies in MM

Multiple studies have examined the role of consolidation and/or maintenance following HDC-ASCT to eliminate residual disease in MM. IFN-*α* was evaluated in 2 major studies and showed improved PFS, however, with substantial side effects and no survival benefit [30, 31]. Glucocorticoids were compared to IFNs and produced similar remission rates, but reinduction at relapse was more successful in those who received post-ASCT IFN [32].

### 4.1. Thalidomide Use after ASCT

Several randomized trials evaluated the use of thalidomide after ASCT [15, 16, 33–38] (see Table 1). Thalidomide's use has been associated with a meaningful improvement in PFS (by approximately 10 months) and EFS. However, the efficacy of thalidomide maintenance was counterbalanced by the significant rate of both acute and long-term side effects leading to discontinuation of the drug in a substantial number of patients ranging between 30–80% by 2 years in different studies [15, 16, 33–35, 37–45].

Due to increased toxicity of maintenance thalidomide, significant improvement in overall survival outcomes was not observed.

**Figure 3:**
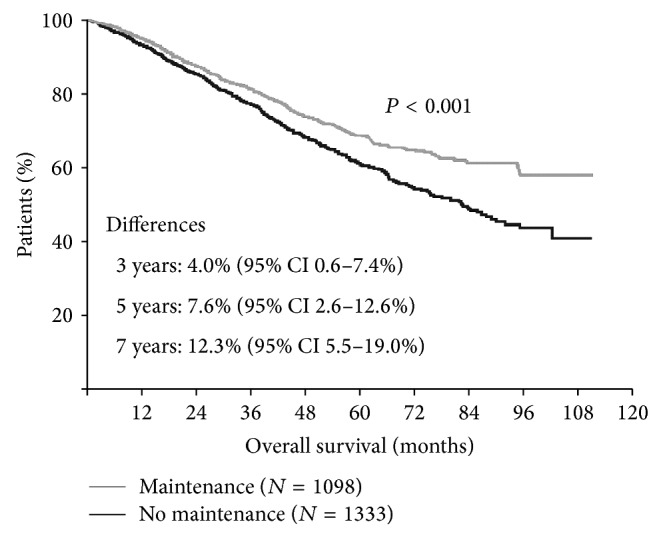
Meta-analysis of thalidomide maintenance showing OS benefit (adapted from [15]).

**Figure 4:**
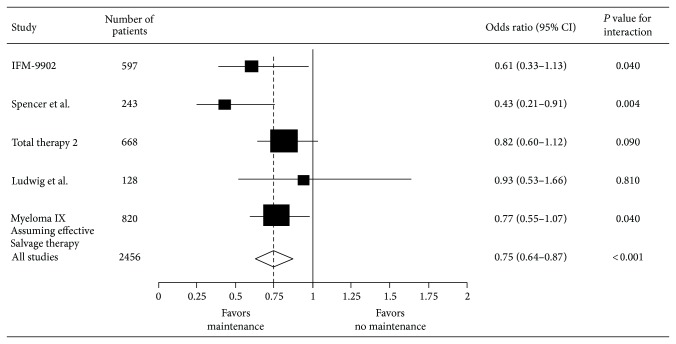
Collective data from thalidomide trials favoring maintenance (adapted from [16]).

In their 2012 meta-analysis, IMWG reviewed 6 randomized trials with a total of 2786 patients evaluating thalidomide use after HDC-ASCT in myeloma [46]. Thalidomide maintenance was associated with significant improvement in PFS (HR 0.65; 95% CI 0.59–0.72) and OS (HR 0.84; 95% CI 0.73–0.97); however, there was considerable heterogeneity among individual trials, likely due to variability in inclusion criteria and salvage treatment of choice at disease progression/relapse (see Figures 3 and 4).

In conclusion, thalidomide use after ASCT appeared to have appeared to be most beneficial in MM with standard-risk disease. It may be more efficacious when combined with a proteasome inhibitor or glucocorticoids, as discussed below [9]. However, in considering such an approach, one should be vigilant about the risk/benefit ratio in individual patients taking into account their comorbidities and residual toxicities from previous treatment, given the considerable discontinuation rate secondary to toxicity noted in different studies.

### 4.2. Lenalidomide Use after ASCT

Two major phase III trials evaluated the role of lenalidomide use after HDC-ASCT in MM and showed improvement in PFS and TTP [47–49]. In the IFM 2005-02 trial, Attal et al. evaluated lenalidomide after ASCT in 614 patients where all study subjects received 2 cycles of lenalidomide consolidation and then got randomized to either lenalidomide maintenance arm (10 mg/day for 3 months and then 15 mg/day) or placebo until disease progression, intolerable side effects, or death [47]. However, at a median of 32 months, maintenance was discontinued when an interim analysis showed increased risk of second primary malignancies (SPMs) with lenalidomide maintenance. At a median follow-up of 45 months, lenalidomide maintenance was associated with improvement in median PFS (41 months versus 23 months in the placebo arm, HR 0.50, *P* < 0.001). However, OS rate at 5 years from diagnosis was similar in both arms.

In comparison, CALGB-100104 trial that evaluated lenalidomide maintenance after ASCT did not include a consolidation phase and it permitted crossover [48]. 460 patients were randomized, after achieving at least stable disease after ASCT, to either lenalidomide maintenance (10 mg/day for 3 months and then 15 mg/day) or placebo until disease progression, intolerance, or death. Interim analysis at 4 years showed significant improvement in TTP (42 months in lenalidomide arm versus 27 months in the placebo arm; *P* < 0.001).

A subgroup analysis showed that patients treated with lenalidomide induction therapy had significantly longer survival if they received lenalidomide maintenance, compared to those who received a placebo. The same analysis also showed that lenalidomide maintenance did not provide a survival advantage for patients who were treated with thalidomide induction therapy, patients who had elevated *β*-2 microglobulin levels, or patients who achieved a CR prior to the start of maintenance therapy or placebo.

Based on these results, the study was unblinded and 82 out of 128 patients in the placebo arm crossed over to the lenalidomide maintenance arm. Dr. McCarthy updated the results of CALGB-100104 trial in 2013 at the International Myeloma Workshop in Japan. Intention-to-treat analysis conducted at a median follow-up of 48 months after crossover showed median TTP of 50 months in the lenalidomide maintenance arm versus 27 months in the placebo arm. OS benefit was maintained at 48 months (median OS not reached in the lenalidomide maintenance arm versus 73 months in the placebo group, *P* = 0.008). Interestingly, OS analysis of patients crossing over from placebo to lenalidomide within 6–12 months of randomization showed significant benefit from lenalidomide maintenance [49].

The difference in survival results between the CALGB and IFM trials may be due to differences in induction consolidation and maintenance therapies.

In the IFM trial, one half got VAD and one quarter got augmented with DICEP, 20% of patients got two transplants, and maintenance was discontinued at a median of 32 months from the start. Longer follow-up and additional studies might clarify the differences in results.

Most recently, Palumbo et al. reported results of the open-label, randomized, phase III study comparing melphalan 200 mg/m^2^ followed by ASCT versus melphalan-prednisone-lenalidomide (MPR). This study also compared lenalidomide maintenance to no maintenance after high-dose melphalan or MPR consolidation. In comparison to no maintenance, lenalidomide maintenance significantly reduced the risk of progression independently of previous induction/consolidation regimen (PFS 41.9 versus 21.6 months, *P* < 0.001) with a trend towards improved 3-year OS rates in the lenalidomide maintenance arm (88% versus 79.2%, *P* = 0.14). Response rates improved during maintenance therapy in both high-dose melphalan and MPR arms. Interestingly, despite a similar CR rate, PFS improved in those who received high-dose melphalan in comparison to MPR chemotherapy. One possible explanation stated by authors is that response was assessed with standard laboratory tests rather than MRD detection by immunophenotypic or molecular techniques which may have revealed subtle differences in response between the two groups [50]. The concept of PFS2, defined as the time from initial randomization to time of objective disease progression after next-line therapy or death from any cause, was recently proposed as a surrogate to OS, particularly in trials evaluating maintenance in MM. This is due to the fact that effective salvage therapies are likely to be available at relapse which is thought to be the main reason for the lack of any statistically significant survival advantage noted across the trials addressing this topic [51].

#### 4.2.1. Second Primary Malignancies (SPMs) after Lenalidomide Maintenance

Enthusiasm for lenalidomide maintenance after HDC-ASCT in MM was subdued by the concern for increased risk of SPMs reported in both IFM and CALGB trials. Interim analysis of IFM trial showed increased risk of SPMs (3.1 versus 1.2 per 100 patients per year) in the lenalidomide maintenance arm [47]. Based on this, lenalidomide maintenance was halted. CALGB trial reported risk of SPMs of 12% in the maintenance versus 6% in the placebo arm (*P* = 0.034) [29]. However, in the CALGB trial maintenance was continued and, of notice, no new cases of SPMs were reported with longer follow-up [49].

The incidence of SPMs following lenalidomide exposure was reviewed in a recently published meta-analysis of 7 randomized trials including 2620 patients treated with lenalidomide versus 598 patients treated with no lenalidomide exposure [52]. Risk of SPMs at 5 years was 6.9% versus 4.8% (HR 1.1; 95% CI 1.03–2.34) which was statistically significant for second primary hematologic malignancies only. This increased risk is thought to be driven by treatment strategies combining lenalidomide with oral melphalan, suggesting that alkylator-free alternatives might be a better combination when using lenalidomide for myeloma patients.

On the other hand, acute myeloid leukemia (AML) and myelodysplasia (MDS) were reported in high frequency in untreated patients with monoclonal gammopathy of undetermined significance (MGUS) [53]. This suggests that hematopoietic stem cell or microenvironmental defect may be leading to the increased risk of hematological malignancies rather than the effect of chemotherapy exposure alone.

In conclusion, data with lenalidomide maintenance after ASCT is favorable. However, the optimal duration of lenalidomide maintenance is still unclear. Lenalidomide provides the most benefit in those who fail to achieve CR or very good partial response (VGPR), by IMWG criteria, after ASCT.

### 4.3. Bortezomib Use after ASCT

The use of bortezomib in the post-ASCT setting in myeloma was evaluated in multiple randomized trials as consolidation, maintenance and in combination with other agents [39, 54, 55]. In the Nordic Myeloma Study Group trial, single-agent bortezomib consolidation given as 20 doses after ASCT resulted in 7-month improvement in PFS compared to placebo (*P* = 0.007); however, no OS benefit was seen. This approach seemed to be most beneficial for patients achieving less than a VGPR after induction and ASCT [54].

HOVON-65 trial evaluated bortezomib given during induction and during maintenance in 827 newly diagnosed myeloma patients [39]. Patients receiving bortezomib had improved PFS compared to those who received nonbortezomib induction regimens (*P* = 0.006). There was a trend towards improvement in 5-year OS survival rates (61% with bortezomib regimens versus 55% in nonbortezomib arm) but this did not reach statistical significance (*P* = 0.07). Bortezomib maintenance significantly improved nCR/CR rate from 31% to 49%, which was found, in a landmark analysis, to be associated with better PFS and OS at 12 months. However, in this trial, no random assignment for maintenance therapy was performed; and, therefore, the effect of that cannot be independently assessed. Furthermore, the major part of the difference in nCR/CR rates between the two groups was observed after induction and ASCT favoring the bortezomib arm. Equivalent upgrade of response was noted with either bortezomib or thalidomide maintenance. Nonetheless, maintenance treatment with bortezomib was much better tolerated than thalidomide maintenance, with fewer patients stopping treatment prematurely. Subgroup analysis showed that the superior outcomes with bortezomib were predominantly accomplished in patients with high-risk disease and myeloma-related renal failure or those with del(17p) and del(13q) [39]. In patients with increased serum creatinine, both median PFS (30 versus 13 months, *P* = 0.004) and OS (54 versus 21 months, *P* < 0.001) were significantly improved in the bortezomib-containing arm compared to those who did not receive bortezomib. In patients with normal serum creatinine, PFS remained superior in the bortezomib arm, whereas OS was similar in both groups. Patients with abnormal FISH results for del(13q14), t(4; 14), and del(17p13) were compared with patients without the abnormality. PFS was worse in all patients with del(13q14) regardless of the treatment arm whereas OS in patient with del(13q14) was similar to those who did not carry the deletion. Of note, OS in patients with the deletion was significantly better in the bortezomib arm in comparison to the nonbortezomib arm. In patients with del(17p13), both PFS (median 22 versus 12 months, *P* = 0.01) and OS (median > 54 months versus 24 months, *P* = 0.003) were significantly better in the bortezomib arm. The presence of t(4; 14) was associated with worse outcomes compared to patients without this translocation. There was a trend towards better outcomes in patients with t(4; 14) who received bortezomib-containing regimen; however, this did not reach statistical significance. This leads us to the conclusion that the use of bortezomib in the post-ASCT setting can potentially overcome the adverse effects of abnormal cytogenetics, a subgroup that has always been associated with inferior outcomes.

### 4.4. Combination Regimens after ASCT

Trials in the recent years focused on combination regimens for consolidation/maintenance after ASCT. The Spanish PETHEMA study was a 3-arm randomized trial that evaluated bortezomib/thalidomide maintenance after ASCT versus single-agent IFN or single-agent thalidomide for a period of 3 years [55]. After a median follow-up of 24 months from maintenance initiation, PFS was significantly longer in bortezomib/thalidomide arm (78%) compared with thalidomide arm (63%) or IFN arm (49%), with no relevant difference in side effects except for higher incidence of neuropathy in the combination arm. Interestingly, the improvement in PFS was primarily seen in patients with low-risk cytogenetics, contrary to what was reported by HOVON-65 trial. This discrepancy might be in part due to different bortezomib dosing and duration (52 doses over 2 years in HOVON-65 versus 48 doses over 3 years in PETHEMA trial).

**Figure 5:**
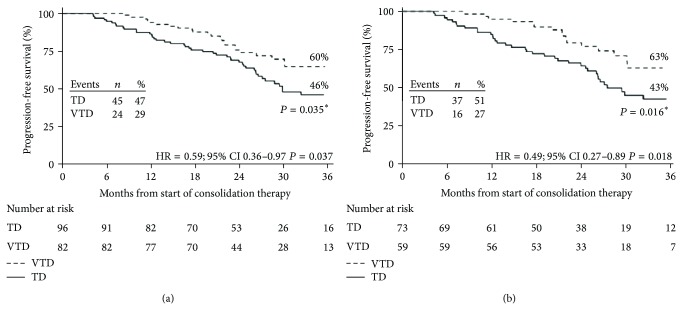
Kaplan-Meier curves for PFS from the landmark of starting consolidation therapy according to the presence or absence of cytogenetic abnormalities. The figure shows PFS for patients with no cytogenetic abnormality or with del(13q) positivity but lack of t(4; 14) and del(17p) or t(4; 14) and/or del(17p) positivity which received VTD consolidation therapy (a) or TD consolidation therapy (b). ^*^ *P* value according to log-rank test (adapted from [17]).

Cavo et al. evaluated the combination bortezomib/thalidomide/dexamethasone (VTD) versus thalidomide/dexamethasone (TD) as consolidation therapy after ASCT in 474 newly diagnosed myeloma patients [17]. After consolidation, CR (60.6% versus 46.6%) and nCR (73.1% versus 60.9%) rates were significantly higher for VTD arm versus TD arm. Notably, VTD consolidation significantly increased CR/nCR rates compared to pretransplant rates, whereas TD did not. With a median follow-up of 30.4 months from initiation of consolidation, 3-year PFS was significantly longer for the VTD group versus TD group (60% versus 48%). Furthermore, PFS curves in the VTD arm were almost superimposable regardless of the presence or absence of cytogenetic abnormality, whereas patients who received TD consolidation and carried abnormal cytogenetics had significantly worse outcomes (Figure 5).

**Figure 6:**
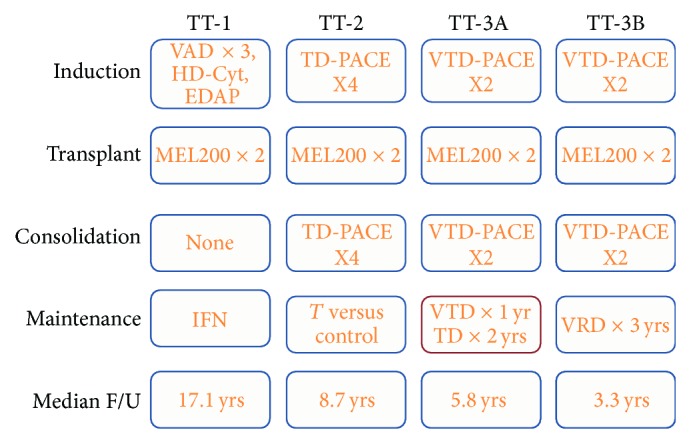
It summarizes the series of Total therapy trials. VAD: vincristine, doxorubicin, and dexamethasone; HD-Cyt: high-dose cyclophosphamide; EDAP: etoposide, dexamethasone, cytarabine, and cisplatin; TD-PACE: thalidomide, dexamethasone, cisplatin, doxorubicin, cyclophosphamide, and etoposide; VTD: bortezomib, thalidomide, and dexamethasone; VRD: bortezomib, lenalidomide, and dexamethasone.

Total therapy (TT) trials are series of studies conducted by the Arkansas Myeloma Group utilizing all active antimyeloma agents upfront to achieve a maximal tumor cytoreduction and thereby increase the frequency and duration of CR, with the goal of extending PFS and OS (see Figure 6).

The introduction of bortezomib in the post-ASCT setting started with TT-3A (VTD-PACE for 2 cycles as consolidation followed by either VTD for 1 year or TD for 2 years). The upfront addition of bortezomib resulted in improved outcomes, compared with TT-2 trials that randomized patients upfront to receive/not receive thalidomide as part of induction, consolidation, and maintenance. To validate these findings along with bortezomib pharmacogenomic data, a successor trial TT-3B enrolled another 177 patients. TT-3A and TT-3B were identical in design, except for the fact that the maintenance phase of TT-3B included VRD (using lenalidomide instead of thalidomide) and was continued for 3 years rather than 1 year in TT-3A (Figure 6). Comparing results of TT-3A and TT-3B, the difference between OS (87% in TT-3B versus 85% in TT-3A) and EFS (83% in TT-3B versus 80% in TT-3A) at 2 years was not statistically significant [56, 57]. When examined in the context of GEP-defined risk, TT-3A and TT-3B result curves were superimposable for both low-risk and high-risk groups. However, more patients with GEP-defined high risk were included in TT-3B versus TT-3A (22% versus 15%, *P* = 0.038). Despite more adverse features in TT-3B and overall higher-risk population, all outcomes (OS, EFS, and CR duration) were similar in the 2 protocols suggesting that 3-year maintenance with a bortezomib-containing regimen is superior to shorter maintenance, along with the reportedly more effective immunomodulatory effect of lenalidomide compared to thalidomide used in TT-3A [56–58]. Furthermore, according to multivariate analysis, deletion TP53 conferred inferior OS and EFS in TT-2 but not in TT-3. The major difference between TT-2 and TT-3 is the addition of bortezomib in induction, consolidation, and maintenance phases. This provides further evidence that the use of proteasome inhibitors in all treatment phases can potentially negate the adverse consequences of deletion TP53, likely through a synergistic mechanism [59].

Most recently, Nooka et al. reported results of a single-arm trial evaluating consolidation and maintenance therapy with lenalidomide, bortezomib, and dexamethasone (RVD) in patients with high-risk cytogenetics, defined as del TP53, del(1p), t(4; 14), or t(14; 16). Patients received different induction regimens followed by ASCT and subsequently RVD maintenance for 3 years followed by lenalidomide until disease progression. Following initiation of RVD maintenance, 51% of patients achieved sCR with 96% achieving at least VGPR as best response. Median PFS for all patients was 32 months and 3-year OS of 93% was reported [60].

Furthermore, in their recent phase II IFM study, Roussel et al. reported high-quality response and favorable tolerability. This phase II trial included 31 newly diagnosed MM patients who were eligible for ASCT. Overall, 27% of patients were classified as having high-risk chromosomal abnormalities based on either having a del(17p) or t(4; 14) abnormality, determined by FISH. Patients received three cycles of RVD and then proceeded to ASCT. Two months after recovery of blood counts, patients who had not progressed received consolidation therapy consisting of two cycles of RVD. Patient subsequently received maintenance therapy with continuous lenalidomide (Revlimid) for one year. Responses deepened significantly after ASCT and consolidation therapy in comparison to responses at the end of induction: 40% reaching sCR versus 10%; 58% of patients were MRD-negative versus 16%. Responses also improved further for some patients during maintenance therapy. After all treatment sequences, 68% of patients had achieved MRD negativity. At a median follow-up of 39 months, the estimated 3-year PFS and OS were 77% and 100%, respectively [61]. None of the patients who had achieved MRD negativity relapsed within three years of diagnosis. Of note, PFS was significantly lower at 23% in patients who had never reached MRD negativity. The most common side effect during lenalidomide maintenance therapy was low blood cell counts.

### 4.5. Maintenance Using Immunotherapeutics and Future Modalities

Dendritic cell vaccination, an example of this approach, works by induction of idiotype-specific T- and B-cell response that can stimulate the body's own immune system to fight and eradicate myeloma cells following administration of idiotype-protein pulsed dendritic cells. Although this seems to be a safe strategy, it has not shown any effects on survival and, to date, remains experimental [62–64].

Other forms of immunotherapy include the monoclonal antibody Elotuzumab (anti-CS1) and Daratumumab (anti-CD38) that have activity both as single agents and in combination with other novel therapeutics. Novel proteasome inhibitors such as Carfilzomib, Marizomib, Ixazomib, and Oprozomib may provide better outcomes in the future. Antibodies to various myeloma cell markers, such as CD40, CD56, CD74, IL-6, TRAIL, and RANKL, combined with proteasome inhibitors, conventional chemotherapy, and/or immunomodulatory agents, may serve as maintenance targets for future research with an ultimate goal of significantly improving OS or potentially curing multiple myeloma [27, 65].

## 5. Summary and Recommendations

Over the last decade, the trials mentioned above have led to markedly improved outcomes in myeloma and answered multiple questions regarding optimal induction regimens as well as highlighting the importance of consolidation/maintenance after ASCT in the era of novel agents. While considering consolidation/maintenance strategies after HDC-ASCT, one needs to take into careful consideration the cost and toxicity involved with the different strategies in order to improve outcomes. The use of sensitive techniques to detect MRD provides a stratification tool to guide further treatment following ASCT.

Patients with low-risk disease and normal cytogenetics, who achieve CR or sCR (per IMWG criteria) after ASCT, especially if this excellent response can be confirmed by MRD negativity, may forgo further therapy after ASCT. These patients will need to be closely followed for evidence of relapse. Patients with high-risk cytogenetics and those with low-/standard-risk cytogenetics who achieve less than a CR should be considered for maintenance treatment. Average time to start consolidation/maintenance is typically 60–100 days after ASCT.

Nooka et al. have reported excellent outcomes for patients with high-risk cytogenetics using combination RVD followed by lenalidomide maintenance. However, this needs further validation in a phase II/III trial design to confirm these superior results. Roussel regimen (2 cycles of RVD followed by lenalidomide maintenance) is promising particularly in the setting of less than a CR with HDC-ASCT.

Our institutional approach for high-risk cytogenetics and low-/standard-risk cytogenetic patients who are still MRD positive after HDC-ASCT is an approach similar to Roussel regimen's with continuation of RVD beyond 2 cycles until achievement of MRD negativity followed by lenalidomide maintenance as long as tolerated or until progression.

## Figures and Tables

**Table 1:** Thalidomide trials in the post-ASCT setting.

Study (authors)	*N*	Maintenance phase study arms	EFS	PFS	OS
IFM 99-02 (Attal et al.) AM [33]	597	A: no maintenance B: pamidronate C: pamidronate/thalidomide (400 mg/d)	A: 36% B: 37% C: 52% (*P* < 0.009)		A: 77% B: 74% C: 87% (*P* < 0.04)

TT-2^*^ (Barlogie et al.) AM [34]	668	A: thalidomide 100 mg/d × 1 year → 50 mg/d until PD B: no maintenance	A: 6 years B: 4.1 years (*P* = 0.001)		A: 57% B: 44% (*P* = 0.09)

HOVON-50 (Lokhorst et al.) AM [35]	556	A: VAD induction → IFN-*α* maintenance B: TAD induction → thalidomide maintenance	A: 22 months B: 34 months (*P* < 0.001)	A: 25 months B: 35 months (*P* < 0.001)	A: 60 months B: 73 months (*P* = 0.77)

MRC myeloma IX intensive group (Morgan et al.) AM [15]	493	A: thalidomide (50–100 mg/d) B: no maintenance	A: 30 months B: 27 months (*P* = 0.003)		A: 75 months B: 80 months (*P* = 0.26)

BMT CTN-0102 (Krishnan et al.) AM [16]	436	A: dexamethasone/thalidomide (200 mg/d) B: no maintenance		A: 49% B: 43% (*P* = 0.08)	A: 80% B: 81% (*P* = 0.82)

(Maiolino et al.) AM [36]	108	A: dexamethasone/thalidomide (200 mg/d) B: dexamethasone		A: 64% B: 30% (*P* = 0.002)	A: 85% B: 70% (*P* = 0.27)

ALLG MM-6 (Spencer et al.) AM [37]	269	A: prednisolone/thalidomide 100–200 mg/d × 12 months B: prednisolone		A: 42% B: 23% (*P* < 0.001)	A: 86% B: 75% (*P* = 0.004)

NCIC CTG MY10 (Stewart et al.) AM [38]	332	A: prednisone/thalidomide (200 mg/d) B: no maintenance		A: 28 months B: 17 months (*P* < 0.0001)	A: 68% B: 60% (*P* = 0.21)

^*^Both arms in TT-2 received the same 4 induction cycles followed by double ASCT and 4 cycles of consolidation (Figure 6). Thalidomide was given in arm A at a dose of 400 mg/d during induction, 100 mg/d during ASCT, and 200 mg/d during consolidation.
